# Miscellany

**Published:** 1901-10

**Authors:** 


### Miscellany.

A Test for Beeswax.—Beeswax is not only grossly adulterated, but entirely substituted with ceresin colored with an analine dye. The latter has the odor and general appearance of true beeswax, but the color is generally brighter and more brilliant than that of true wax. On keeping, the color may disappear entirely, leaving the wax a dingy white.

The simplest test for beeswax is to take its specific gravity. This is easiest accomplished by melting a little of the sample and dropping it into alcohol so as to form globules. These should be allowed to stand in the alcohol overnight, and then just enough cold water added to the alcohol to cause the wax globules to float indifferently in the mixture, with either rising or sinking quickly. To be accurate, the temperature of the mixture should be just 15° C. (59° F.) when this is accomplished. The alcoholic solution is then poured off and its specific gravity, which is the same as that of the sample of wax, is ascertained by means of a specific gravity flask. A wax which will pass this test can be regarded as pure if the specific gravity corresponds to that given in the Pharmacopœia.

White wax is less likely to be adulterated than yellow wax, but it always contains a small portion of foreign fat, which is usually rancid.—Wilbur L. Scoville, *Australian Journal of Pharmacy.*

---

Method of making Removable Facings.—Dr. A. E. Peck has an original method of making removable facings similar to the Mason. He takes an ordinary facing with the pins above one another and uses gold, 30-gauge, for backing, bending the pins down tightly. He raises the gold on either side of the pins to an angle of forty-five degrees, fills the V-shape with solder, and finishes to the desired thickness, after which he burnishes a piece of pure gold to this backing and stiffens it with solder. It can then be used the same as a Mason tooth.—*Dental Review.*

---

To wet Wheels while grinding Teeth.—I find a simple and easy device for wetting a wheel while grinding teeth is to affix a small piece of sponge by means of a rubber band to the middle finger. This is easily held against the corundum wheel. It is a simple device, yet one of the handiest for the purpose which I have ever seen or tried. The quantity of water can be regulated just right with it.—C. C. Jordan, *Dental Hints.*

---

Mounting Crowns and Bridges.—Were it possible to successfully manipulate gutta-percha, the advantages of ease of removal, insolubility, and the cushion-like effect afforded would render it practically ideal; but, unfortunately, it is difficult to successfully manipulate in extensive cases, and requires considerable time and much effort in single crowns.

I usually give preference to cement, but always observe the precaution of coating the interior of the crown and post with a thin film of chloro-percha or gum of shellac to facilitate removal in case of necessity.—Dr. H. F. Goslee, *Dental Register.*

---

Home-made Porcelain Inlay Furnace.—It is made of sheet-iron with joints riveted, and in size is five and one-half inches high, four inches deep, three and one-half inches wide, and fastened to a base of iron.

The opening for the muffle is one inch high and one and one-half inches wide, and admits a double muffle, which is three and one-half inches long, one and three-eighths inches wide, and seven-eighths of an inch high. Each muffle is made of nickel, twenty-four to twenty-six gauge, and formed by beating and malleting to a pattern of wood. In the bottom is an opening for the blow-pipe. At the top there is an opening or outlet for the flame and gases after circulating around the muffle. At the lower part of the opening for the muffle is a small shelf to facilitate the handling of the work. The furnace is lined with a mixture of fire-clay, powdered asbestos, and liquid silex. While this is in a plastic state, the muffle is inserted to embed the end in the lining, which, when hard, holds it firmly in place and prevents the influx of gas.

The blow-pipe used is of his own design, similar to the Fletcher blast. Ash's high-fusing body can be baked in from five to seven minutes; the low-fusing body in from one to three minutes. The approximate cost of the furnace is from one to two dollars.

The above furnace was designed by Dr. Wessels and exhibited at the Pennsylvania Association of Dental Surgeons on April 9, 1901.—*Dental Brief.*

---

Setting Bridges with Gutta-Percha instead of Cement.—At the Minnesota State Dental Society, held at Minneapolis, Dr. A. E. Peck illustrated a new method of setting bridges with S. S. White soft gutta-percha instead of cement. He used stopping slightly heated and placed it on the pin; then inserted the crown or bridge. The same was removed and the surplus stopping trimmed off. A drop of cement was then placed on the pin and the crown or bridge driven to place. The advantage in using the S. S. White soft gutta-percha is that it yields readily to heat.—*Dental Review.*

---

To secure Good Edges to Gold Fillings when using a Matrix.—I wish to mention one thing in connection with a matrix, which I have used several times very advantageously, and I believe others have used it successfully. Fold over the matrix before placing it in position several layers of non-cohesive gold, so that it covers and extends beyond the margins of the cavity. It is necessary to have sufficient space to permit its being slid up between the teeth. When in position, it is wedged tightly against the cavity. Then insert an instrument between the gold and the matrix about the centre of that side of the cavity and force the gold carefully back into the cavity, so as to cover the floor and walls as far as it will go. This makes the gold continuous over and beyond all margins and leaves quite a quantity of gold between the matrix and the margins, held there firmly in place. You can then proceed in any way that you may desire in filling the cavity with cohesive or non-cohesive gold, and you are sure, with ordinary care in introducing and packing the remainder of the gold, that all margins are safely covered. Dr. R. B. Tuller, *Dental Review.*

---

Simple Electro-Gold Plating Apparatus.—A one-and-a-half-volt dry cell, that is ordinarily used for electric bells about the house, will generate enough electricity; two pieces of copper wire (the ends of which are attached to the poles of the cell) long enough to reach the vessel containing the plating solution; a porcelain or glass dish or cup that will hold about a half-pint or a pint, three inches in diameter, with parallel sides, is the outfit. In the dish is placed a fully saturated solution of cyanide of potassium in water. A piece of pure gold is attached to the end of the wire from the positive pole, while the metal to be plated is attached to the end of the wire from the negative pole. Both the gold and the metal to be plated are now immersed in the solution, but not allowed to come in contact with each other. If the plating is going on, there will be an activity about the metal upon which the gold is being deposited.

There is a good deal of detail and experience needed before plating can be successfully done, even after the appliance for doing it is perfected.

In recommending the plating bath to be a saturated solution of cyanide of potassium, we are quite well aware that a gold chloride solution in cyanide of potassium is advised, but in practice it is found unnecessary. The gold chloride solution is expensive and easily spoiled. The cyanide solution seems to answer every purpose.—*Dominion Dental Journal.*

---

Cocaine Solution for Hypersensitive Dentine.—The *Dental Brief* publishes the following from the *Dental Register:*

I have four solutions, all of which I can depend upon,—one with alcohol, one with chloroform, one with alcohol and chloroform mixed, and one with a fifty per cent. solution of sulphuric acid. Cocaine mixed with all these acts beautifully. With the fifty per cent. solution of sulphuric acid it makes a very oily looking mixture, and is one of the best means I know of for the reduction of hypersensitiveness of dentine. The sulphuric acid solution should be neutralized before filling.

